# Transitional care for formerly incarcerated persons with HIV: protocol for a realist review

**DOI:** 10.1186/s13643-017-0428-4

**Published:** 2017-02-13

**Authors:** Jenkin Tsang, Sharmistha Mishra, Janet Rowe, Patricia O’Campo, Carolyn Ziegler, Fiona G. Kouyoumdjian, Flora I. Matheson, Ahmed M. Bayoumi, Shatabdy Zahid, Tony Antoniou

**Affiliations:** 1 The Li Ka Shing Knowledge Institute, St. Michael’s Hospital, Toronto, ON Canada; 2 Department of Medicine, University of Toronto, Toronto, ON Canada; 3 Centre for Urban Health Solutions, St. Michael’s Hospital, Toronto, ON Canada; 4 Prisoners with HIV/AIDS Support Action Network, Toronto, ON Canada; 5 Dalla Lana School of Public Health, University of Toronto, Toronto, ON Canada; 6 Department of Family Medicine, McMaster University, Hamilton, ON Canada; 7 Division of General Internal Medicine, St. Michael’s Hospital, Toronto, ON Canada; 8 Institute of Health Policy, Management and Evaluation, University of Toronto, Toronto, Ontario Canada; 9 Department of Family and Community Medicine, St. Michael’s Hospital and University of Toronto, 410 Sherbourne Street, 4th Floor, Toronto, ON M4X 1K2 Canada

**Keywords:** HIV, Prisoners, Intervention, Realist review, Post-prison

## Abstract

**Background:**

Little is known about the mechanisms that influence the success or failure of programs to facilitate re-engagement with health and social services for formerly incarcerated persons with HIV. This review aims to identify how interventions to address such transitions work, for whom and under what circumstances.

**Methods:**

We will use realist review methodology to conduct our analysis. We will systematically search electronic databases and grey literature for English language qualitative and quantitative studies of interventions. Two investigators will independently screen citations and full-text articles, abstract data, appraise study quality and synthesize the literature. Data analysis will include identifying context-mechanism-outcome configurations, exploring and comparing patterns in these configurations, making comparisons across contexts and developing explanatory frameworks.

**Discussion:**

This review will identify mechanisms that influence the success or failure of transition interventions for formerly incarcerated individuals with HIV. The findings will be integrated with those from complementary qualitative and quantitative studies to inform future interventions.

**Systematic review registration:**

PROSPERO CRD42016040054

**Electronic supplementary material:**

The online version of this article (doi:10.1186/s13643-017-0428-4) contains supplementary material, which is available to authorized users.

## Background

The transition from prison to community is challenging for formerly incarcerated persons with HIV [1]. Most incarcerated individuals with HIV eventually return to their communities, yet programs to facilitate re-engagement with health and social services at the time of transition are often not available [1, 2]. Such transition intervention programs are important because people who are incarcerated are two- to 50-fold more likely to be HIV-positive than the general population [3–7]. Among people with HIV, formerly incarcerated persons have a higher prevalence of mental illness, substance use, and homelessness [1, 8, 9]. Consequently, people with HIV who are transitioning from prison to community settings are at risk of negative outcomes, including interruption of antiretroviral therapy, harmful substance use and discontinuities in primary health care, as well as high rates of emergency department use related to overdose and mental illness and the inability to access social services [1, 10–17]. The transition to community is also associated with an increase in high-risk sexual behaviours [18–20]. Thus, interventions that help individuals to successfully bridge the transition from prison to community offer an opportunity to improve individual health and public health and optimize the efficient delivery of health services.

Little is known about the program features, contextual factors and mechanisms that influence the success or failure of transition interventional programs for formerly incarcerated individuals with HIV. Previous work provides insights into the composition of such programs [21–30]. However, an in-depth understanding of the mechanisms by which these interventions cause change to occur in specific contexts and for different populations is important for effectively adapting and implementing programs across settings.

We will conduct a realist review of studies describing programs that address the transition to community from incarceration for people with HIV. Realist review is a form of knowledge synthesis focused on explaining how interventions work by uncovering the mechanisms that cause change to occur within specific contexts [31]. Our specific research question is ‘What is known about how programs that address transition for formerly incarcerated individuals with HIV work, for whom they work, and the mechanisms contributing to their success or failure?’ In this protocol paper, we outline our initial program theory and describe the specific steps that will be undertaken according to the preferred reporting items for systematic review and meta-analysis protocols (PRISMA-P) 2015 statement [32] (Additional file 1).

## Methods

### Realism

Realist synthesis is a theory-driven method of literature review which applies the logic and philosophy of critical realism to knowledge synthesis [31, 33, 34]. In critical realism, a distinction is made between the real (causal powers or mechanisms of objects and agents), the actual (what happens if and when those mechanisms are activated) and the empirical (events which are experienced or observed) domains of reality [35, 36]. A key ontological tenet of realism is therefore the stratification of reality into those events which can be experienced and observed, and the invisible and context-sensitive generative mechanisms behind them. When applied to program evaluation and knowledge synthesis, realist inquiry posits that mechanisms (M) that cause change or outcomes (O) to occur within specific contexts (C) are embedded within interventions [31, 33, 34]. Mechanisms include the beliefs, values, desires and cognitive or emotional reasoning of participants and stakeholders who receive or deliver interventions. The mechanisms are usually hidden and therefore not directly observable. Instead, mechanisms are identified by asking what it is about an intervention that generates change and examining how individuals’ reactions (such as engagement or resistance) to the resources offered by interventions lead to specific outcomes [34]. Context refers to any condition that activates or modifies the mechanism. Examples of context include social, cultural, historical and institutional norms that constrain or facilitate agency, trust-building processes, geographic location and funding sources. Outcomes are the expected or unexpected products of the interaction between mechanisms and context.

The goals of realist synthesis are to uncover context-mechanism-outcome (CMO) configurations that are the basis for understanding how and why interventions work [31, 33, 34]. For example, a CMO configuration may include the following: a community with a high rate of injection drug overdose among formerly incarcerated persons with HIV (C) implements an addiction program for these individuals, but enrollment is low (O) because participants are fearful of re-incarceration if they are seen engaging with the program (M).

### Approach

#### Step 1: articulating an initial program theory and scope of the review

The first step in a realist review is to develop a program theory of existing interventions. Program theory refers to ‘the mechanisms that intervene between the delivery of the program … and the occurrence of outcomes of interest. It focuses on participants’ responses to [the] program’ [37]. For our review, we developed a preliminary program theory that included the activities and strategies offered to participants by the intervention, the participant responses to these strategies (e.g. linkage to care), salient contextual information and the theorized intervening mechanisms between program strategies and participant responses that trigger change.

**Fig. 1:**
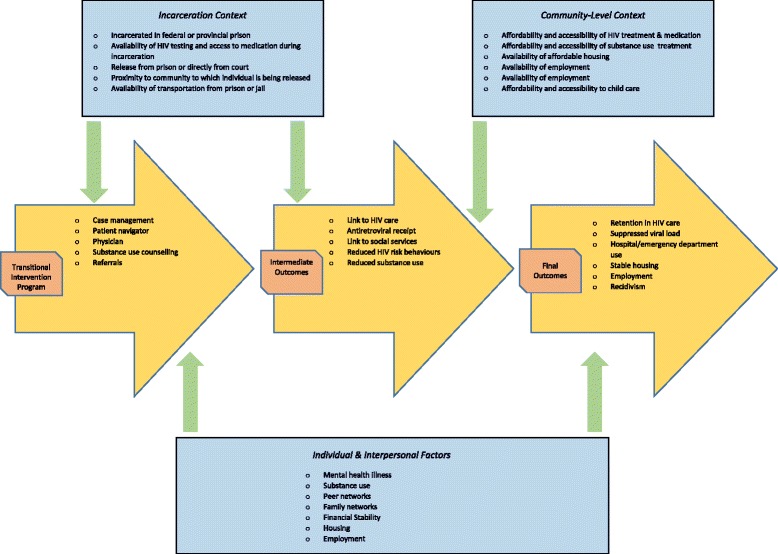
Preliminary program theory

Our preliminary program theory and scope of the review were informed by literature examining barriers to post-release linkage to care for formerly incarcerated individuals with HIV, consultations with community members with lived experience of transitioning from prison to community and conversations with staff of provincial AIDS Service Organizations involved in the provision of care to these individuals. From these activities, we learned that formerly incarcerated individuals with HIV prioritize meeting basic needs over HIV-specific health needs in the immediate post-release period, a challenge that is often magnified by mental health illness, substance use and unstable social networks [38–41]. In addition, we identified contextual differences between releases from provincial jails versus federal penitentiaries. Releases from provincial jails are often precipitous and occur without planning and often directly from court. In contrast, federal prisons may offer pre-release planning, but because penitentiaries may be located at a considerable distance from an individual’s home community, distance and travel impose additional barriers to post-release engagement with care. In addition, our initial review of program descriptions indicates that existing interventions that address transition to community living include peer support workers, case management, patient navigation and referrals to community agencies [21–30]. Based on these findings, we conceptualized a preliminary program theory encapsulating the transition intervention program, ‘intermediate’ outcomes of linkage to health and social support services and decrease in post-release HIV risk behaviours, and ‘final’ outcomes, including retention in HIV care, recidivism and suppression of viral load (Fig. 1).

The current review will begin by examining the relationship between transition intervention programs and the intermediate outcomes. We selected these outcomes as the initial focus for two reasons. First, the final outcomes are at least one step removed from transition intervention programs, which are designed primarily to facilitate linkage to care and services required in the immediate post-release period as well as provide risk reduction and substance use counselling. Second, even if the proximal outcomes are achieved, the final outcomes may involve mechanisms that are unrelated to the transition intervention program. For example, successful retention in HIV care following linkage may be related less to the transition intervention program and more to mechanisms specific to the clinic environment, such as perceived stigma or discrimination. However, because of the public health implications of the final outcomes, we will stay open to including and reviewing separately studies that examine the relationship between transition intervention programs and final outcomes. Revisions to the breadth and depth of the review are common and expected in realist syntheses [34].

As part of developing our program theory, we theorized that mechanisms linking the various components of the transition intervention programs to the intermediate outcomes would be related to various dimensions of these interventions, including their content, staffing, setting(s), extent of community consultation and initial ‘buy-in’. Based on our preliminary literature review and consultations, we classified candidate mechanisms into the following broad categories: ‘relatedness’, ‘safety’, ‘understanding’, ‘motivation’, ‘congruence’ and ‘endorsement’. The relatedness mechanisms are said to be operating when engagement or resistance to the intervention is influenced by participants’ reactions to the staff delivering the intervention, or vice versa. Safety mechanisms are those that refer to participants’ comfort or concerns related to the intervention, such as fears of disclosure or stigma. Understanding and motivation mechanisms are hypothesized to operate when intervention content is culturally or linguistically adapted for the target population being served and seeks to increase education and/or help-seeking behaviour on the part of participants. Congruence mechanisms occur when participants respond positively or negatively to intervention activities based on how well they align with their post-release needs. Finally, endorsement mechanisms are those that are in operation when participant engagement is influenced by the extent of community consultation involved in determining the nature of programs offered by the intervention. We speculated that each intervention component may be linked with more than one outcome and that, consequently, multiple mechanisms may exist within each strategy. For example, in a community where a high proportion of formerly incarcerated persons with HIV are of a particular ethno-racial group (context), an intervention where case managers are of similar ethnic background as clients may engender trust (mechanism–relatedness) and increase knowledge (mechanism–understanding) of how to link with HIV medical care (outcome). Conversely, it is possible that such an intervention may be unsuccessful (outcome) if participants resist in engaging with the case managers because of fear of being recognized by individuals from within their own community (mechanism–safety). Similarly, where interventions are staffed by peer workers with a lived experience of transitioning from prison to community (context), the shared history with participants may increase staff motivation (mechanism–motivation) to facilitate client linkage to care, resulting in actions such as following up with clients who missed medical appointments (outcome). This level of investment on the part of the staff may further increase client trust (mechanism–relatedness) in the intervention. However, individuals may resist such interventions if, for example, peer support was not a post-release priority (mechanism–congruence) for the population of formerly incarcerated people with HIV served by the intervention (context), a mismatch generated by a lack of consultation with the community prior to program implementation (mechanism–endorsement). For interventions where client contact is initiated by a case manager in a community setting to which the individual is returning (e.g. home of a family member), linkage to care may be facilitated by receipt of the intervention in a setting that is familiar (mechanism–safety) to the individual. In contrast, fear of disclosure of HIV status (mechanism–safety) to family members or other close social networks may result in resistance to interventions which are embedded within the community, particularly in smaller urban centers or rural settings (context). At this stage, we consider these mechanisms to be candidate mechanisms only, which will be subsequently tested against the literature and further refined and revised based on the review.

#### Step 2: literature search

We will develop a systematic search strategy in consultation with an information scientist. We will search the following electronic bibliographic databases from 1980 to the time of the review: MEDLINE, EMBASE, Cumulative Index to Nursing and Allied Health Literature (CINAHL), Cochrane Collaboration Library, Campbell Collaboration Library, PsycINFO, Web of Science, Social Science Abstracts, Social Services Abstracts, Social Work Abstracts, Sociological Abstracts, Criminal Justice Abstracts and the International Bibliography of the Social Sciences (see Appendix for draft MEDLINE search). The search strategy will be reviewed by another experienced information scientist using the Canadian Agency for Drugs and Technology in Health Peer Review of Electronic Search Strategies checklist [42]. We will also search for relevant grey literature through GreyNet International and websites of associations that work with incarcerated persons with HIV; websites of national corrections associations in Europe, North America and Australia; the World Health Organization; dissertations; and research collections held by Canadian groups such as the Homeless Hub, the National Aboriginal Health Organization and the Wellesley Institute. We will include quantitative and qualitative studies published in the English language that describe interventions to facilitate post-release linkage to health and social services for formerly incarcerated persons with HIV, and we will exclude abstracts, letters, editorials, commentaries and case studies. We will not exclude studies based on country or setting in which interventions were described.

#### Step 3: study selection

We will import all citations obtained using the search strategy into Covidence^TM^ for de-duplication and study screening and selection. Study selection will proceed according to the three stages as described below:

#### Stage 1: title and abstract screening

Two independent reviewers will screen the titles and abstracts to eliminate studies that (i) do not include formerly incarcerated individuals with HIV and (ii) do not describe an intervention to facilitate post-release engagement with health and social services. Differences in judgment will be resolved by a third reviewer. When abstracts are not available or there is uncertainty about inclusion, we will obtain the full article, which two independent reviewers will screen for inclusion.

#### Stage 2: data abstraction, management and appraisal

The investigators involved in study selection will independently extract data from eligible studies using a customized data extraction form. The data extraction form will be pilot-tested with five to ten studies to ensure clarity and congruence of extracted data and revised accordingly. Data to be extracted will include study characteristics (e.g. study date, methodology), the program elements that are described, characteristics of the study population, theoretical or conceptual frameworks employed, contextual factors described (e.g. geographic, social, cultural, legislative), type of intervention (e.g. behavioural, structural), whether the authors describe possible mechanisms that could lead to the outcome(s) and outcomes examined. We will use the ‘Guidance for the Assessment of Context and Implementation in Health Technology Assessments (HTA) and Systematic Reviews of Complex Interventions: The Context and Implementation of Complex Interventions (CICI) Framework’ to facilitate extraction of contextual and implementation factors [43]. The two investigators involved in data abstraction will meet regularly to discuss and resolve differences in independently extracted information from the same articles.

We will appraise study quality using the approach outlined in the RAMESES publication standards for realist syntheses [44]. Specifically, articles will be appraised based on their relevance to the review and their rigor (i.e. was the information generated using credible and trustworthy methods). To examine study credibility, we will use appraisal tools relevant to different study designs. Specifically, we will use the Cochrane Risk of Bias tool for randomized controlled trials [45], the Newcastle-Ottawa Scale for observational studies [46], the Mixed-Method Appraisal Tool for mixed-method studies [47], and the substantive approach to evaluating qualitative research outlined by Eakin and Mykhaloviskiy [48]. We will not exclude studies using these tools but expect that some articles may be excluded if it is decided that they cannot contribute to our understanding of how interventions facilitate post-release linkage to care work. Disagreements between the two reviewers will be resolved by a third reviewer.

#### Step 4: synthesis and interpretation

We will use the realist approaches of intra-program and inter-context comparison to synthesize the literature [34]. In the first stage of synthesis, we will conduct intra-program analysis by summarizing and mapping visually the life cycle of each intervention, including a description of its various components, the chain of implementation steps, who initiates the intervention, groups targeted by the intervention and descriptions of stakeholders. We will contact authors for information regarding implementation and context if these data are not found in the publication. We will use the conceptual maps and summaries to generate CMO configurations for each study, describing how contextual factors interact with mechanisms to produce outcomes. Next, we will group studies with similar interventions together and explore patterns within the various CMO configurations using the conceptual tools of realist synthesis, including situating (e.g. ‘this mechanism in context A, that one in context B’), reconciling (identifying differences which explain apparently contradictory findings), juxtaposing (using process data from one study to make sense of outcome patterns noted in another) and consolidating (building multi-faceted explanations) [34]. In the second stage of synthesis, we will conduct inter-context analysis, by making comparisons across contexts [34]. Specifically, we will examine whether mechanisms are consistent in producing similar outcomes in different populations of formerly incarcerated persons with HIV, across different geographic locations and in different incarceration settings (e.g. state/provincial versus federal). In this way, we will formulate statements of what works, for whom and under which circumstances. In the final stage, we will configure these statements into an explanatory framework (or frameworks) encompassing individual, interpersonal, and institutional/systems-level components. The CMO configurations, statements and frameworks will be shared with community members to review consistency with lived experience and to solicit feedback regarding clarity and adaptations to the framework(s). We will use the findings of the synthesis process and consultations to refine our original program theory and prepare a final report.

## Discussion

We developed a realist review protocol to understand the mechanisms that may drive the success or failure of programs designed to support the transition of formerly incarcerated persons with HIV into communities. Our realist review will provide important information regarding the mechanisms and contexts that explain the success or failure of interventions to link formerly incarcerated individuals with HIV to health and social services in the period immediately following the release from jail or prison. However, several potential limitations merit emphasis. First, research studies are generally not written to be read with a realist lens, and it is therefore possible that many studies will not report contextual or implementation factors. We will attempt to address the gaps in literature by contacting authors. Second, publication bias is possible, in that only studies reporting successful interventions might be reported. We will attempt to mitigate this bias by contacting experts in the field of HIV and prison health, who may be aware of unpublished studies in this field. Finally, by prioritizing our scope to intermediate outcomes, we may be unable to examine whether associations exist between transitional intervention programs and final outcomes. However, limitations on the scope of realist reviews are common and necessary given the complexity of interventions and the varied contexts in which they operate.

We will use several knowledge dissemination strategies to reach target stakeholders. First, we will produce a report that integrates and discusses the findings of the rapid realist review and the complementary studies using the Canadian Health Services Research Foundation 1:3:25 (i.e. one page of take home messages, three-page executive summary, 25-page report) format [49]. Second, we will generate plain-language fact sheets that can be freely accessed by community members at AIDS Service Organizations. Third, we will publish our findings in a peer-reviewed journal. Fourth, we will present the results of our research to reach our diverse audience of stakeholders and community members, including seminars, hospital rounds, and relevant Canadian and international meetings. Finally, to facilitate users’ ability to identify our results, we will make the report, fact sheets, database of studies in the rapid realist review, manuscripts and presentations available to stakeholders for posting on their organizations’ websites.

The results of this review will be integrated with findings from a series of complementary qualitative and quantitative studies to inform the development and evaluation of a transition intervention program for formerly incarcerated individuals with HIV.

## Appendix

Ovid MEDLINE search strategy

Database: Epub Ahead of Print, In-Process & Other Non-Indexed Citations, Ovid MEDLINE(R) Daily and Ovid MEDLINE(R) <1946 to Present>

Search strategy:

1. Prisoners/
2. Criminals/
3. Prisons/
4. (correctional system or felon* or imprison* or incarcerat* or jail* or offender* or prison* or convict* or inmate* or correctional facilit* or criminal justice system* or criminal justice setting* or corrections or correctional setting*).tw,kw.
5. 1 or 2 or 3 or 4
6. “continuity of patient care”/or patient transfer/or transitional care/
7. Aftercare/
8. (post or ex or release* or former* or reentry or reintegrat* or reenter or re-entry or re-integrat* or re-enter or transition*).ti.
9. link* to care.tw,kw.
10. 6 or 7 or 8 or 9
11. 5 and 10
12. (after prison or parole* or probation or community reentry or community re-entry or ex-convict* or ex-inmate* or ex-offender* or ex-prisoner* or former convict* or former inmate* or former offender* or former prisoner* or formerly incarcerated or offender* reenter* or offender* re-enter* or offender* reentry or offender* re-entry or offender* reintegrat* or offender* re-integrat* or offender* release or out of jail or postincarceration or post-incarceration or postprison or post-prison or postrelease or post-release or prison to community or prison to society or prisoner* reenter* or prisoner* reentry or prisoner* reintegrat* or prisoner* re-enter* or prisoner* re-entry or prisoner* re-integrat* or prisoner* release* or release* from prison or release* from correction* or return to communit*).tw,kw.
13. 11 or 12
14. exp HIV Infections/
15. exp HIV/
16. HIV Long-Term Survivors/
17. exp Anti-Retroviral Agents/
18. human immunodeficiency virus.tw,kw.
19. human immune-deficiency virus.tw,kw.
20. (human immun* and deficiency virus).tw,kw.
21. acquired immunodeficiency syndrome.tw,kw.
22. acquired immuno-deficiency syndrome.tw,kw.
23. acquired immune-deficiency syndrome.tw,kw.
24. (acquired immun* and deficiency syndrome).tw,kw.
25. “HIV/AIDS”.mp.
26. (hiv or hiv-1* or hiv-2* or hiv1 or hiv2).tw,kw.
27. Antiretroviral drugs.tw,kw.
28. Antiretroviral therapy.tw,kw.
29. Anti-retroviral therapy.tw,kw.
30. Antiretroviral treatment.tw,kw.
31. or/14-30
32. 13 and 31
33. limit 32 to english language
34. limit 33 to yr = “1980 -Current”
35. remove duplicates from 34

## Additional file

Additional file 1: PRISMA-P 2015 checklist. (DOCX 36 kb)

## Abbreviations

CMO: Context-mechanism-outcome
HIV: Human immunodeficiency virus

## References

[1] Springer SA, Altice FL, Greifinger R (2007). Improving the care for HIV-infected prisoners: an integrated prison-release health model. Public Health Behind Bars.

[2] Solomon L, Montague BT, Beckwith CG, Baillargeon J, Costa M, Dumont D (2014). Survey finds that many prisons and jails have room to improve HIV testing and coordination of postrelease treatment. Health Aff (Millwood).

[3] Calzavara L, Ramuscak N, Burchell AN, Swantee C, Myers T, Ford P (2007). Prevalence of HIV and hepatitis C virus infections among inmates of Ontario remand facilities. CMAJ.

[4] United Nations Office on Drugs and Crime (UNODC) (2007). HIV and prisons in sub-Saharan Africa: opportunities for action.

[5] Centers for Disease Control and Prevention. HIV among incarcerated populations. Available at: http://www.cdc.gov/hiv/group/correctional.html. Accessed 16 Nov 2016.

[6] Correctional Service Canada. Infectious disease surveillance in Canadian federal penitentiaries: 2007-2008 pre-release report. Available at: http://www.csc-scc.gc.ca/text/pblct/infdscfp-2007-08/index-eng.shtml. Accessed 16 Nov 2016.

[7] Poulin C, Alary M, Lambert G, Godin G, Landry S (2007). Gagnon, et al. Prevalence of HIV and hepatitis C virus infections among inmates of Quebec provincial prisons. CMAJ.

[8] Haley DF, Golin CE, Farel CE, Wohl DA, Scheyett AM, Garrett JJ (2014). Multilevel challenges to engagement in HIV care after prison release: a theory-informed qualitative study comparing prisoners’ perspectives before and after community reentry. BMC Public Health.

[9] Travis J, Solomon AL, Waul M. From prison to home: the dimensions and consequences of prisoner reentry. Available at: http://research.urban.org/UploadedPDF/from_prison_to_home.pdf. Accessed 17 Nov 2016.

[10] Palepu A, Tyndall M, Chan K, Wood E, Montaner JS, Hogg RS (2004). Initiating highly active antiretroviral therapy and continuity of HIV care: the impact of incarceration and prison release on adherence and HIV treatment outcomes. Antivir Ther.

[11] Springer SA, Freidland GH, Doros G, Pesanti E, Altice FL (2007). Antiretroviral treatment regimen outcomes among HIV-infected prisoners. HIV Clin Trials.

[12] Springer SA, Wohl DA, Golin CE, Tien HC, Stewart P, Kaplan AH (2005). Effect of release from prison and re-incarceration on the viral loads of HIV-infected individuals. Public Health Rep.

[13] Springer SA, Pesanti E, Hodges J, Macura T, Doros G, Altice F (2004). Effectiveness of antiretroviral therapy among HIV-infected prisoners: re-incarceration and the lack of sustained benefits and release to the community. Clin Infect Dis.

[14] Baillargeon J, Marx R, Pendo W, Wu ZH, Wells K, Pollock BH (2008). Accessing antiretroviral therapy following release from jail. JAMA.

[15] Althoff AL, Zelenev A, Meyer JP, Fu J, Brown SE, Vagenas P (2013). Correlates of retention in HIV care after release from jail: results from a multi-site study. AIDS Behav.

[16] Krishnan A, Wickersham JA, Chitsaz E, Springer SA, Jordan AO, Zaller N (2013). Post-release substance abuse outcomes among HIV-infected jail detainees: results from a multisite study. AIDS Behav.

[17] Meyer JP, Qiu J, Chen NE, Larkin GL, Altice FL (2012). Emergency department use by released prisoners with HIV: an observational longitudinal study. PLoS One.

[18] Clements-Nolle K, Marx R, Pendo M, Loughran E, Estes M, Katz M (2008). Highly active antiretroviral therapy use and HIV transmission risk behaviors among individuals who are HIV infected and were recently released from jail. Am J Public Health.

[19] Stephenson BL, Wohl DA, McKaig R, Golin CE, Shain L, Adamian M (2006). Sexual behaviours of HIV-seropositive men and women following release from prison. Int J STD AIDS.

[20] Adimora AA, Schoenbach VJ, Martinson FEA, Donaldson KH, Stancil TR, Fullilove RE (2003). Concurrent partnerships among rural African Americans with recently reported heterosexually transmitted HIV infection. J Acquir Immune Defic Syndr.

[21] Conklin TJ, Lincoln T, Flanigan TP (1998). A public health model to connect correctional health care with communities. Am J Public Health.

[22] Altice F, Khoshnood K (1997). Transitional case management as a strategy for linking HIV-infected prisoners to community health and social services (Project TLC).

[23] Lincoln T, Kennedy S, Tuthill R, Roberts C, Conklin TJ, Hammett TM (2006). Facilitators and barriers to continuing healthcare after jail: a community-integrated program. J Ambul Care Manage.

[24] Thompson AS, Blankenship KM, Selwyn PA, Khoshnood K, Lopez M, Balacos K (1998). Evaluation of an innovative program to address the health and social service needs of drug-using women with or at risk for HIV infection. J Community Health.

[25] Zaller ND, Holmes L, Dyl AC, Mitty JA, Beckwith CG, Flanigan TP (2008). Linkage to treatment and supportive services among HIV-positive ex-offenders in Project Bridge. J Health Care Poor Underserved.

[26] Ko NY, Liu HY, Lai YY, Pai YH, Ko WC (2013). Case management interventions for HIV-infected individuals. Curr HIV/AIDS Rep.

[27] Draine J, Ahuja D, Altice FL, Arriola KJ, Avery AK, Beckwith CG (2011). Strategies to enhance linkages between care for HIV/AIDS in jail and community settings. AIDS Care.

[28] Laufer FN, Arriola KR, Dawson-Rose CS, Kumaravelu K, Rapposelli KK (2002). From jail to community: innovative strategies to enhance continuity of HIV/AIDS care. Prison J.

[29] Fox AD, Anderson MR, Bartlett G, Valverde J, MacDonald RF, Shapiro LI (2014). A description of an urban transitions clinic serving formerly incarcerated people. J Health Care Poor Underserved.

[30] Springer SA, Spaulding AC, Meyer JP, Altice FL (2011). Public health implications for adequate transitional care for HIV-infected prisoners: five essential components. Clin Infect Dis.

[31] Pawson R, Greenhalgh T, Harvey G, Walshe K (2005). Realist review - a new method of systematic review designed for complex policy interventions. J Health Serv Res Policy.

[32] Moher D, Shamseer L, Clarke M, Gersi D, Liberati A, Petticrew M (2014). Preferred reporting items for systematic review and meta-analysis protocols (PRISMA-P) 2015 statement. Syst Rev.

[33] Pawson R, Tilley N (1997). Realistic evaluation.

[34] Wong G, Westhorp G, Pawson R, Greenhalgh T. Realist synthesis: RAMESES training materials. RAMESES Project, 2013. http://www.ramesesproject.org/media/Realist_reviews_training_materials.pdf. Accessed 16 Nov 2016.

[35] Sayer A (2000). Realism and social science.

[36] Connelly JB (2007). Evaluating complex public health interventions: theory, methods and scope of realist enquiry. J Eval Clin Pract.

[37] Weiss CH (1997). Theory-based evaluation: past, present and future. N Dir Eval.

[38] Fox AD, Anderson MR, Bartlett G, Valverde J, Starrels JL, Cunningham CO (2014). Health outcomes and retention in care following release from prison for patients of an urban post-incarceration transitions clinic. J Health Care Poor Underserved.

[39] Hammett TM, Donahue S, LeRoy L, Montague BT, Rosen DL, Solomon L (2015). Transitions to care in the community for prison releasees with HIV: a qualitative study of facilitators and challenges in two states. J Urban Health.

[40] Harzke AJ, Ross MW, Scott DP (2006). Predictors of post-release primary care utilization among HIV-positive prison inmates: a pilot study. AIDS Care.

[41] Rozanova J, Brown SE, Bhushan A, Marcus R, Altice FL (2015). Effect of social relationships on antiretroviral medication adherence for people living with HIV and substance use disorders and transitioning from prison. Health Justice.

[42] Sampson M, McGowan J, Cogo E, Grimshaw J, Moher D, Lefebve C (2009). An evidence-based practice guideline for the peer review of electronic search strategies. J Clin Epidemiol.

[43] Pfadenhauer L, Rohwer A, Burns J, Booth A, Bakke Lysdahl K, Hofmann B (2016). Guidance for the Assessment of Context and Implementation in Health Technology Assessments (HTA) and Systematic Reviews of Complex Interventions: The Context and Implementation of Complex Interventions (CICI) Framework.

[44] Wong G, Greenhalgh T, Westhorp G, Buckingham J, Pawson R (2013). RAMESES publication standards: realist syntheses. BMC Med.

[45] Higgins JPT, Green S (editors). Cochrane Handbook for Systematic Reviews of Interventions Version 5.1.0 [updated March 2011]. The Cochrane Collaboration, 2011. Available: http://handbook.cochrane.org. Accessed 2 Jan 2017.

[46] Wells G, Shea B, O’Connell D, Peterson J, Welch V, Losos M, et al. The Newcastle-Ottawa Scale (NOS) for assessing the quality of nonrandomised studies in meta-analyses. Ottawa Hospital Research Institute, 2014. Available: http://www.ohri.ca/programs/clinical_epidemiology/oxford.asp. Accessed: 2 Jan 2017.

[47] Pluye P, Gagnon M-P, Griffiths F, Johnson-Lafleur J (2009). A scoring system for appraising mixed methods research, and concomitantly appraising qualitative, quantitative and mixed methods primary studies in mixed studies reviews. Int J Nurs Stud.

[48] Eakin JM, Mykhalovskiy E (2003). Reframing the evaluation of qualitative health research: reflections on a review of appraisal guidelines in the health sciences. J Eval Clin Pract.

[49] The Canadian Health Services Research Foundation (CHSRF) (2009). Communication notes: reader-friendly writing - 1:3:25.

